# Application of an emotional classification model in e-commerce text based on an improved transformer model

**DOI:** 10.1371/journal.pone.0247984

**Published:** 2021-03-05

**Authors:** Xuyang Wang, Yixuan Tong

**Affiliations:** School of Computer and Communication of the Lanzhou University of Technology, Lanzhou City, Gansu Province, China; Hefei University of Technology, CHINA

## Abstract

With the rapid development of the mobile internet, people are becoming more dependent on the internet to express their comments on products or stores; meanwhile, text sentiment classification of these comments has become a research hotspot. In existing methods, it is fairly popular to apply a deep learning method to the text classification task. Aiming at solving information loss, weak context and other problems, this paper makes an improvement based on the transformer model to reduce the difficulty of model training and training time cost and achieve higher overall model recall and accuracy in text sentiment classification. The transformer model replaces the traditional convolutional neural network (CNN) and the recurrent neural network (RNN) and is fully based on the attention mechanism; therefore, the transformer model effectively improves the training speed and reduces training difficulty. This paper selects e-commerce reviews as research objects and applies deep learning theory. First, the text is preprocessed by word vectorization. Then the IN standardized method and the GELUs activation function are applied based on the original model to analyze the emotional tendencies of online users towards stores or products. The experimental results show that our method improves by 9.71%, 6.05%, 5.58% and 5.12% in terms of recall and approaches the peak level of the F1 value in the test model by comparing BiLSTM, Naive Bayesian Model, the serial BiLSTM_CNN model and BiLSTM with an attention mechanism model. Therefore, this finding proves that our method can be used to improve the text sentiment classification accuracy and effectively apply the method to text classification.

## Introduction

With the rapid development of technology and the rapid popularization of the mobile internet in the past few years, many emerging industries have been born, and an increasing number of people are willing to comment on merchants and the products they buy on review platforms, including Dianping.com, Meituan, Eleme, etc. The comment information shows a certain emotional tendency, so analyzing these texts assists with product improvement, user selection, keyword extraction, etc. As artificial intelligence has been rapidly developing in recent years, traditional manual processing is gradually being eliminated, and the wide application of artificial intelligence algorithms has greatly promoted the rapid development of text sentiment classification. Currently, there are basically 3 methods commonly used in the field of text sentiment classification: the semantic-dictionary-based method, the machine-learning-based method and the deep-learning-based method.

Through learning from existing deep learning models, we found that the CNN model can only obtain partial text information when processing text classification. If the distance between 2 words is too long but there is still a dependency between them, the CNN model is unable to detect with good accuracy. The RNN model shows a certain memory ability and can handle long-distance dependencies between words very well. However, RNN has a high cost to train the model, making it too expensive to train. Therefore, in text sentiment classification, existing models still have shortcomings, including relatively low accuracy and recall, a long training time, etc.

Addressing the above problems, we try to optimize the method based on the transformer model to improve the recall rate and reduce the model training time to a certain extent. The accuracy of the data is guaranteed by adding new variables in the multihead attention mechanism. In addition, a new normalization method, instance normalization, is applied to process the data. Finally, the model is optimized with activation functions to improve the ability of the model to perform random regularization, to make it more consistent with the natural process of cognition and to effectively improve its overall performance.

## Related work

Google proposed the transformer model in 2017 [1]. In existing encoding and decoding frameworks, most deep learning frameworks are achieved by CNN or RNN; meanwhile, the transformer model removes traditional CNN and RNN structures and only uses the attention mechanism to achieve certain goals. Therefore, the transformer model is used as an encoding and decoding model based entirely on the attention mechanism. Additionally, because the attention mechanism was introduced by the transformer model, the entire architecture consists of a stacked self-attention and fully connected layer. The context with distant words is combined via an attention mechanism, all words are processed in parallel, and each word notices other words in the sentence in multiple processing steps.

**Fig 1:**
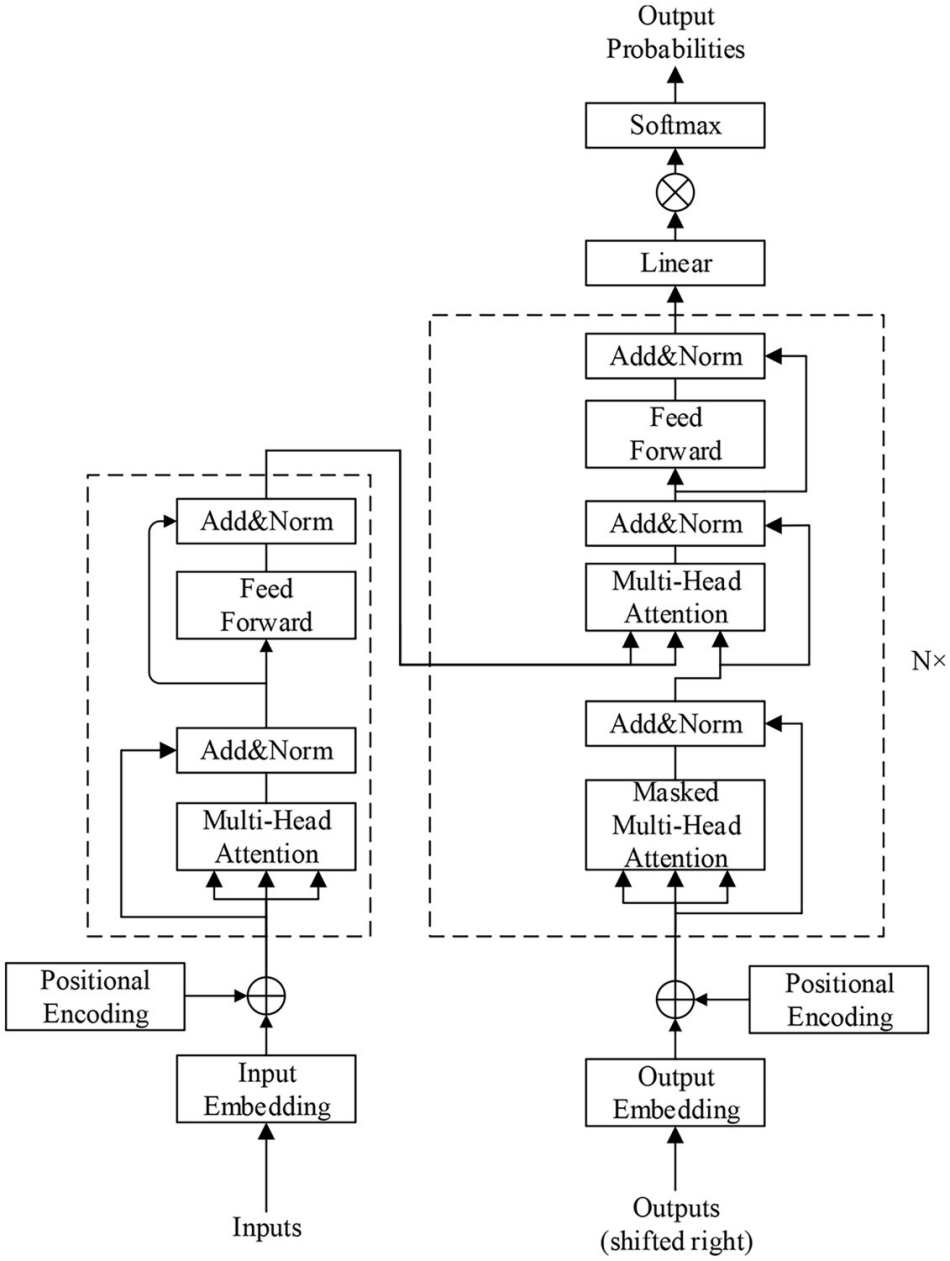
Transformer model diagram. This picture shows the overall structure of the transformer model.

Transformer model is shown in Fig 1 as follow.

The left half of Fig 1 is the encoder, and the right half is the decoder. The encoder is stacked in 6 layers that have the same structure, and each layer can be further divided into 2 sublayers, i.e., multihead attention and feed forward. The decoder is also formed by 6 layers that have the same structure, but the contents of each layer are different from those in the encoder module. The first layer uses multihead attention to calculate the input self-attention. The second layer performs an attention calculation on the data input by the encoder module. The third layer is the feed forward layer. It follows that when executing the decoder module, each layer of the decoder will apply multihead attention on the result obtained by the encoder. By adopting 2 attention mechanisms, scaled dot-product attention and multihead attention, the transformer model greatly improves task performance, parallel processing and trainability.

### Scaled dot-product attention mechanism

Scaled dot-product attention treats the input sequential encode representation as a set of key-value pairs (K, V) and a query Q, where the relationship of K and V is one-to-one. By means of querying every element in Q, each element in K is multiplied to find the inner product. Then, the softmax method is used to calculate the similarity between elements in Q and elements in V. A weighted sum is then used to obtain a new vector. Among them, T represents the matrix transpose, $\frac{1}{\sqrt{d_{k}}}$ is the scaling factor, where dk represents the dimension of the key, and 64 is used by default. And its formula is shown below.

$$\begin{array}{c} \text{Attention}\;\left(Q,K,V\right)=\mathrm{softmax}(\frac{QK^{T}}{\sqrt{d_{k}}})V \end{array}$$ (1)

Scaled dot-product attention adds a dimension with a value of K on the basis of a similarity calculation using a dot product operation, which plays a role in regulating the inner product in case the value sought is too large.

**Fig 2:**
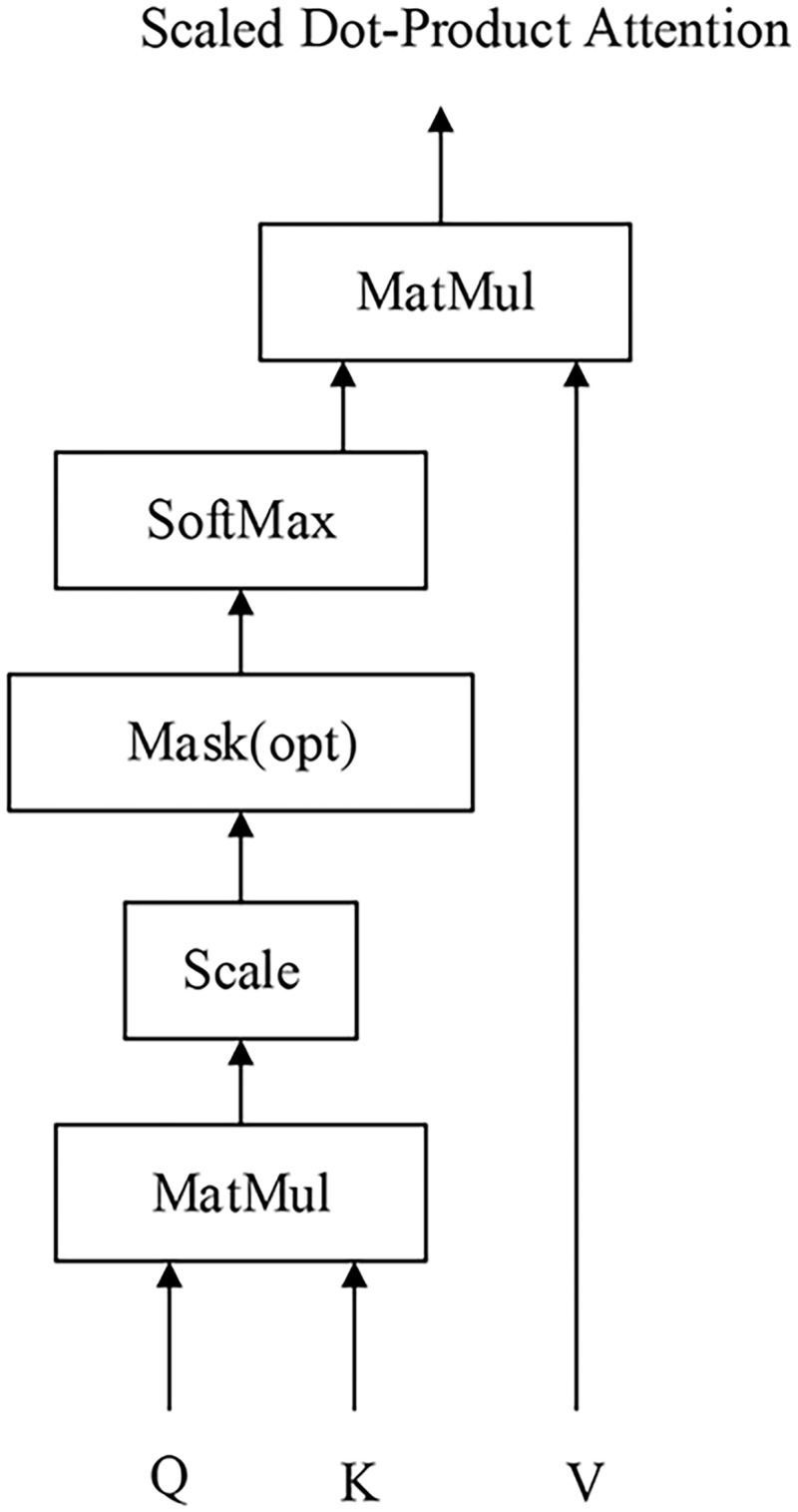
Scaled dot-product attention. This picture introduces the structure of the Scaled dot-product attention mechanism.

Scaled dot-product attention is as shown in Fig 2.

### Multihead attention

**Fig 3:**
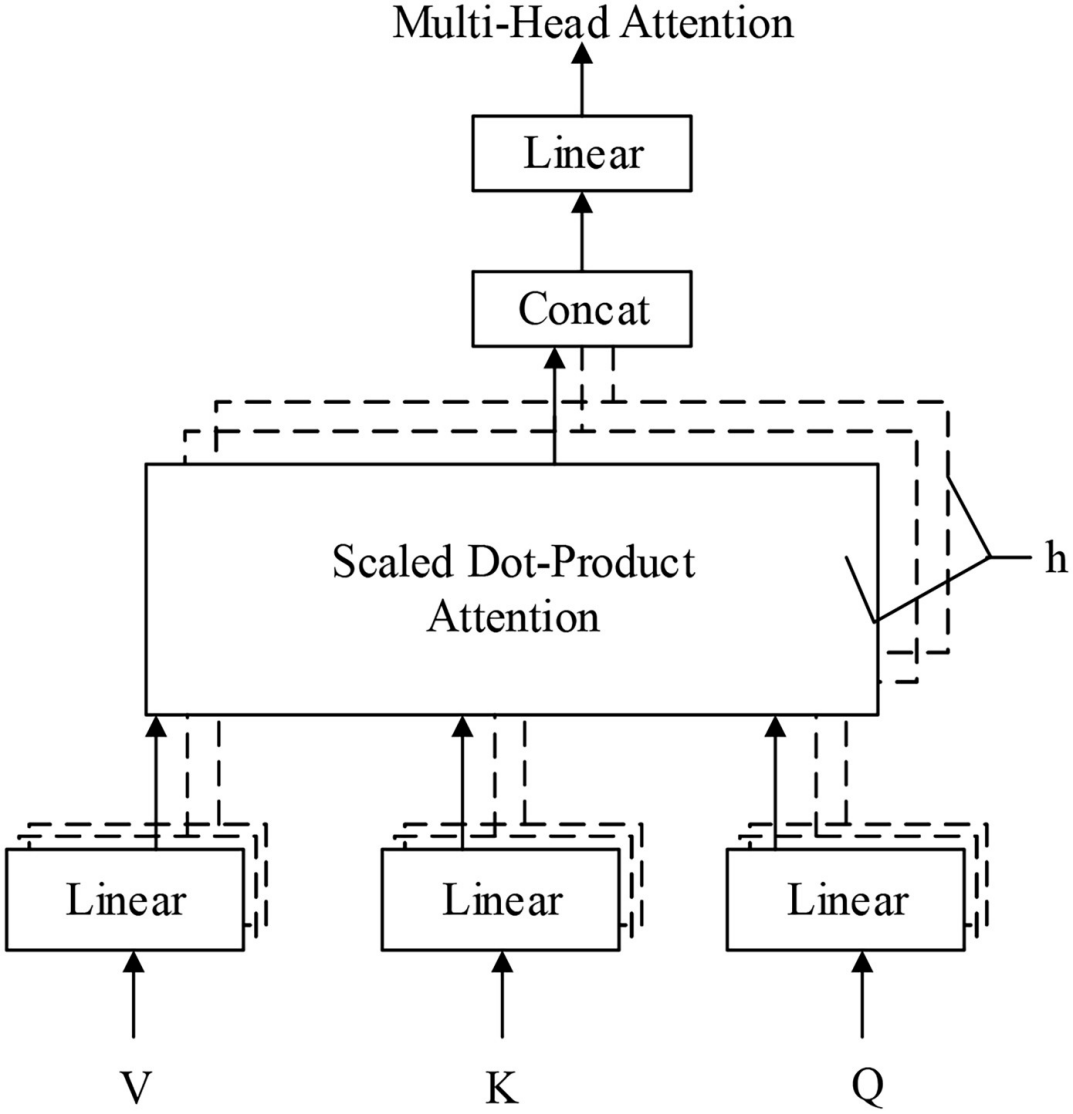
Multi-head attention. This picture introduces the structure of the Multi-Head Attention mechanism.

The structure of multihead attention is as shown in Fig 3 below. Query, key and value first go through a linear transformation. Then the results are input into scaled dot-product attention h times, namely, multihead attention, where each input is treated as a head. Moreover, the parameters W for the linear transformation of each input Q, K and V are different. The results from the h-time scaled dot-product attention are combined; the value is determined after performing a linear transformation as the result of multihead attention. As above, the model is characterized by an h-time calculation instead of merely learning the results of one calculation. The advantage is that this strategy allows the model to learn relevant information in different representation subspaces. The formula of the model is shown below.

$$\begin{array}{c} \text{MultiHead}\;\left(Q,K,V\right)=\text{Concat}\;(head_{1},\ldots ,head_{h}\text{)}W^{o} \end{array}$$ (2)

$$\begin{array}{c} {\text{head}}_{i}=\text{Attention}\;(QW_{i}^{Q},KW_{i}^{K},VW_{i}^{V}) \end{array}$$ (3)

### Layer normalization method

Layer normalization (LN) [2] is applied to normalize the sample. The depth of the transformer model is not fixed, and many static features need to be saved in each training; therefore, if there is a particular order in the calculation process, the subsequent model training will be difficult. The LN method is not required to perform batch training, and normalization operations can be performed within a single piece of data. The LN method performs normalization operations on the inputs to all neurons in a certain layer of deep learning according to the formula below.

$$\begin{array}{c} \mu^{l}=\frac{1}{H}\sum_{i=1}^{H}a_{i}^{l} \end{array}$$ (4)

$$\begin{array}{c} \sigma^{l}=\sqrt{\frac{1}{H}\sum_{i=1}^{H}{\left(a_{i}^{l}-\mu^{l}\right)}^{2}} \end{array}$$ (5)

Moreover, in the LN method, the inputs into neurons of the same layer share the same means and variances, while a different input sample will have different means and variances, which avoids the effects of training caused by insufficient variance and other conditions. In addition, the LN method processes on a single training sample and is independent of other data. Therefore, the LN method can avoid the impact of different data distributions on model training.

### Word2vec

Word2vec was proposed by Tomas in 2013 [3], which makes the original neural network language model with too many parameters and huge computational effort simple and easy. The core of the algorithm is a neural network approach that uses the bag-of-words model CBOW (continuous bag-of-words) or Skip-Gram model to map words into the same coordinate system for learning lexical vectors in a corpus.

The CBOW model, which is a prediction of the current word vector *W*_*t*_ with a known context *W*_(*t*−2)_, *W*_(*t*−1)_, *W*_(*t*+1)_, *W*_(*t*+2)_, learns the objective function of maximizing the log-likelihood function as shown in the following equation.

$$\begin{array}{c} L=\sum_{w=C}\log p\left(w|\text{Context}\left(w\right)\right) \end{array}$$ (6)

Skip-gram model, which is to predict the contextual word vectors with known current words, with the function shown below.

$$\begin{array}{c} L=\sum_{w=C}\log p\left(\text{Context}\;\left(w\right)|w\right) \end{array}$$ (7)

Word2vec is trained using the gradient ascent method, and in order to improve the training performance, both Hierarchical Softmax and Negative Sampling methods are used for solving.

## Model design

**Fig 4:**
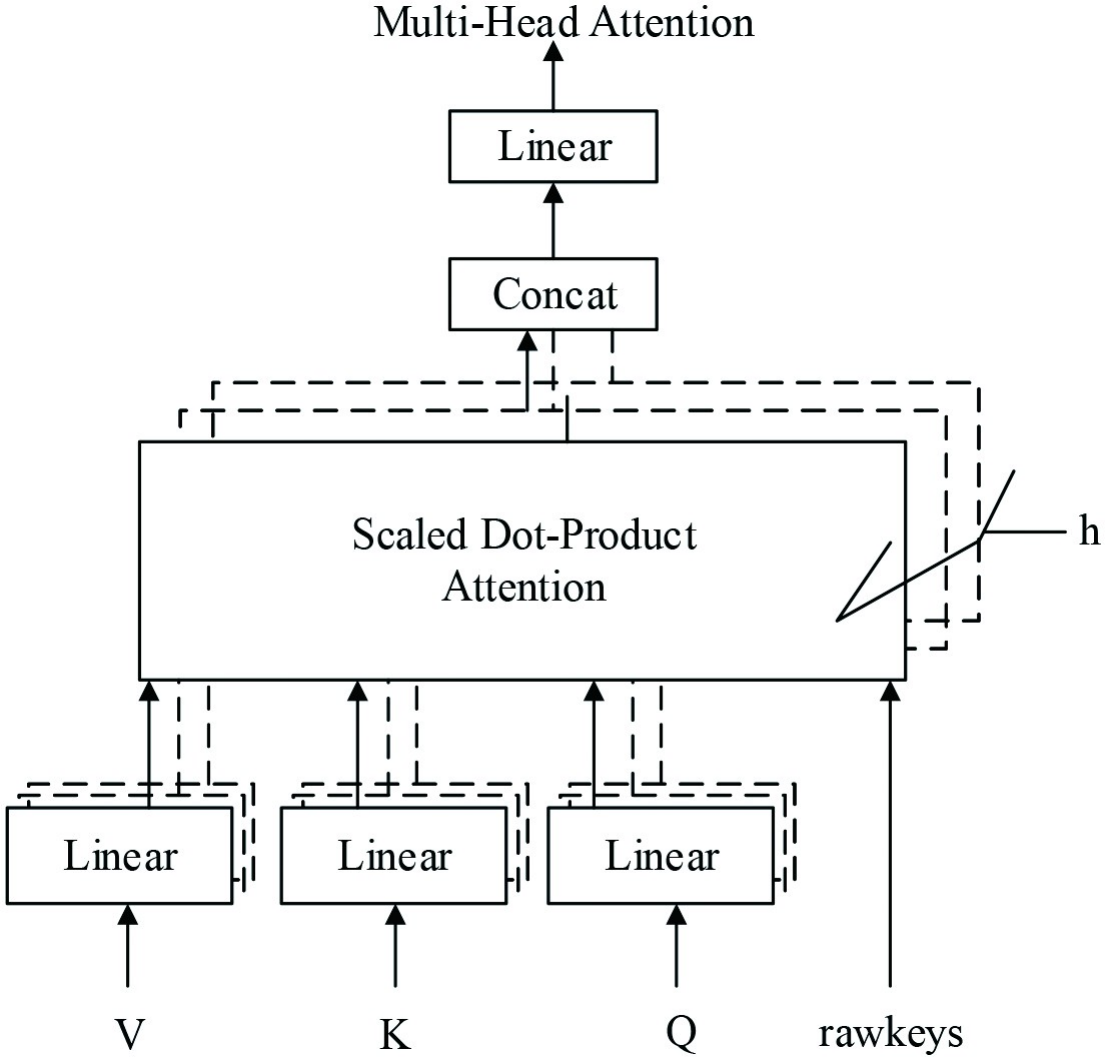
Multi-head attention model with rawkeys variable. This picture shows the structure diagram of the multi-head attention model after adding the rawkeys variable.

**Fig 5:**
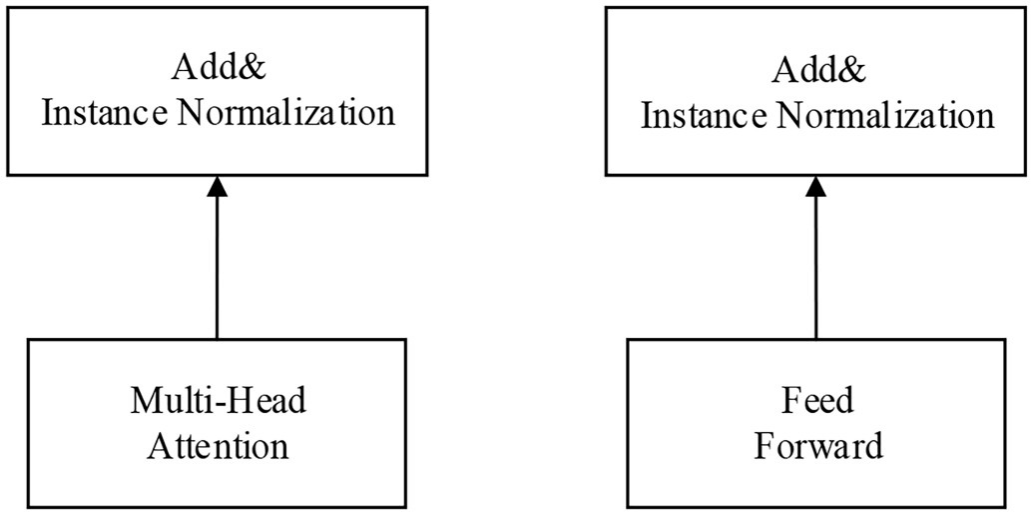
Using the IN method. This picture shows the model structure diagram using the IN standardization method in the final data processing stage.

To increase the recall of text sentiment classification and inspired by DeepMind, this paper proposes a modified transformer model. It is found that in the model based on transformer+softmax, when processing the dataset used in this paper and when the padding is 0, overfitting of the softmax function will occur during optimization. Inspired by rawkey, our model adds a new rawkeys variable to multihead attention and fills the data before they are applied to the softmax function to avoid the condition when the padding is 0. This process benefits the optimization of the softmax function, ensuring the accuracy of the result, as shown in Fig 4. Additionally, this paper introduces a new normalization method, instance normalization (IN), to normalize data. When processing data, it is not affected by channels or batchsize and also guarantees the independence of each text instance, as shown in Fig 5.

Finally, this paper also modifies the original activation function and introduces GELUs. Thus, the model is more consistent with the cognition process in nature by adding random regularization.

### Word vector model

**Fig 6:**
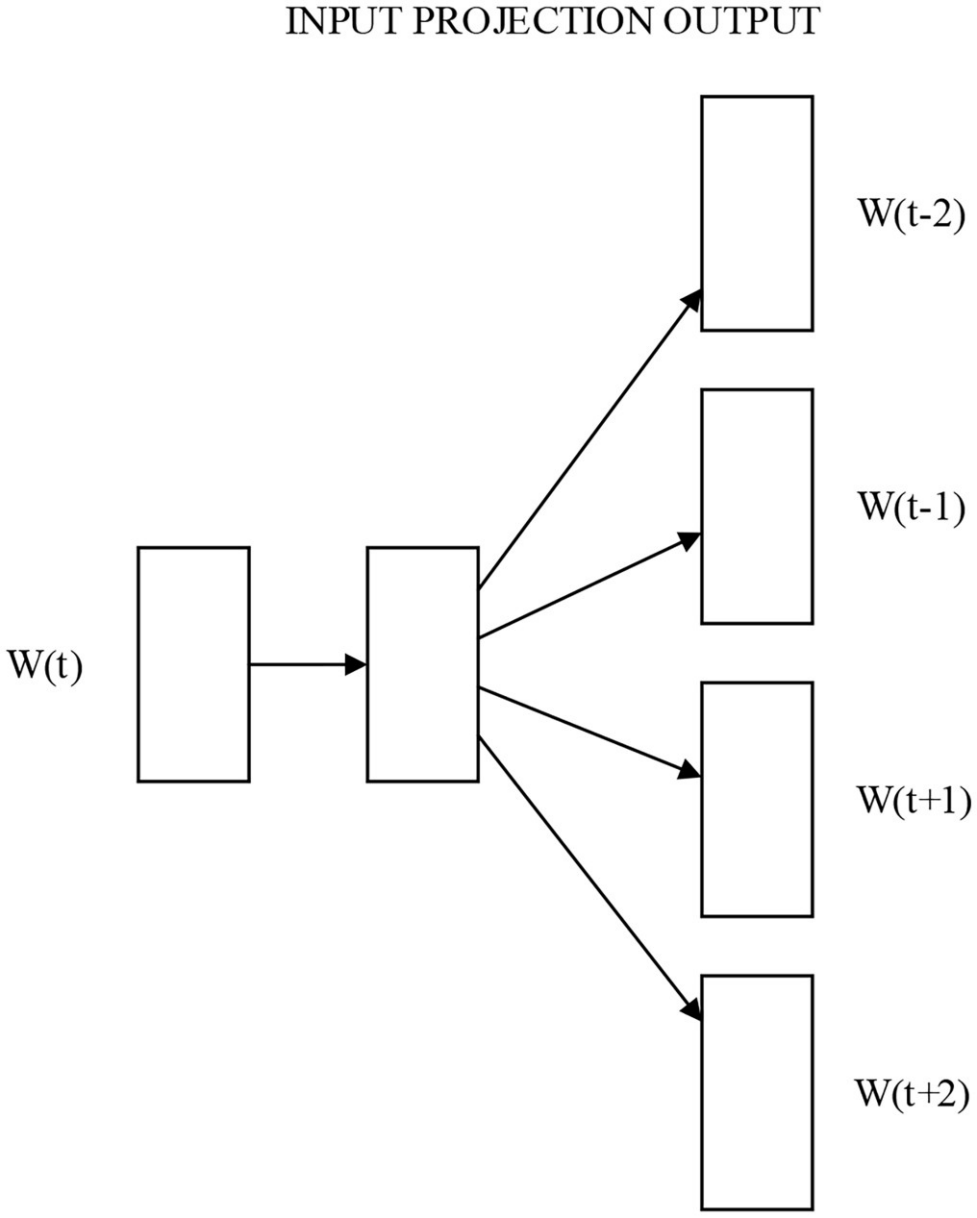
skip-gram model. This picture is a schematic diagram of the model of the skip-gram model.

The word vector model uses the word2vec model [3] for training. Using the skip-gram model in word2vec, the vector dimension is set to 100, the number of iterations is 8 and the training result is saved in the format of bin. In the word embedding layer, the position definition is transmitted by means of a fixed one-hot method and stitched to a word vector [4]. As for the generation of the position vectors, this paper applies sine and cosine to obtain the embedding part of each position. Then parity is used and wrapped with sine and cosine. Subsequently, this paper utilizes batchsize, sequencelen and embeddingsize to construct a 3-dimensional matrix. The skip-gram model is displayed in Fig 6.

### Rawkey variable

This paper adds the rawkey variable to the multihead attention of the original transformer model to calculate the value of the mask. By means of adding rawkey, the variable of keys is added with the value of position embedding, so it does not contain padding with a value of 0. The function is covered with a very small number before application of the softmax function, leading to 0 points as the result. Similarly, if there is no value when filling the query, then it will be covered by 0. The use of masking depends on whether the sum of the query result in the last dimension is equal to 0, so the filled part is added by position embedding and will never be 0.

### Normalization method

In terms of the choice of normalization method, this paper applies a new normalization method, instance normalization [5], to normalize the samples. Compared with the LN used in the original paper, the same mean and variance are shared when inputting into neurons of the same layer, and different means and variances are provided for different input samples to avoid the impact of insufficient variance and other conditions on training neural networks; however, its effect is only average. The underlying reason for its average effect is that, considering the dependency goal of mini-batch, the LN method regards the response values of all neurons in the same layer as the collection range to calculate means and variances. Therefore, when the range of the statistical value is narrowed, its strength is no longer obvious.

However, the IN method picked in this paper is able to avoid this problem. The IN method was first proposed in the field of image processing and was later applied to image pixel normalization. This paper analyzes the theory of IN, utilizes it to normalize textual features, and makes the theory accelerate model convergence while keeping the independence of each text instance. In addition, the normalization of the IN method is not affected by the size of the channel or batch. IN normalizes the data according to the formula below.

$$\begin{array}{c} y_{tijk}=\frac{x_{tijk}-\mu_{ti}}{\sqrt{\sigma_{ti}^{2}+\epsilon }} \end{array}$$ (8)

$$\begin{array}{c} \mu_{ti}=\frac{1}{HW}\sum_{l=1}^{W}\sum_{m=1}^{H}x_{tilm} \end{array}$$ (9)

$$\begin{array}{c} \sigma_{ti}^{2}=\frac{1}{HW}\sum_{l=1}^{W}\sum_{m=1}^{H}{\left(x_{tilm}-mu_{ti}\right)}^{2} \end{array}$$ (10)

### GELUs activation function

For the choice of activation function, the selected GELUs (Gaussian Error Linear Units) is a new activation function proposed by Dan and others in 2018. GELUs is a neural network activation function of high performance, which has been applied successfully in the bert model [6]. Compared to existing activation functions, including ReLU, Sigmoid, etc., the sigmoid function is easily saturated, while the ReLU function lacks random factors [7]. However, in neural network modeling, a very important property of a model is its nonlinearity. Moreover, for the generalization ability of a model, random regularization and other factors need to be introduced. The two factors above are separated. GELUs introduces the idea of random regularization in activation, which is a probabilistic description of the neuron input. From a visual point of view, GELUs is more consistent with the natural cognitive process for which the mathematical expression is as follows:

$$\begin{array}{c} \mathrm{GELUs}(x)=xP(X\leq x)=x\Phi (x) \end{array}$$ (11)

where Φ(x) is the probability function of the normal distribution. If using a simple normal distribution N(0,1), then the mathematical formula of the GELUs(x) for a hypothetic standard normal distribution is calculated, as shown below.

$$\begin{array}{c} \text{GELUs}\left(x\right)=0.5x\;(1+\text{tanh}|\sqrt{\frac{2}{\pi }}(x+0.044715x^{3})|) \end{array}$$ (12)

or

$$\begin{array}{c} x\sigma (1.702x) \end{array}$$ (13)

## Experiment and analysis

### Experimental dataset

**Fig 7:**
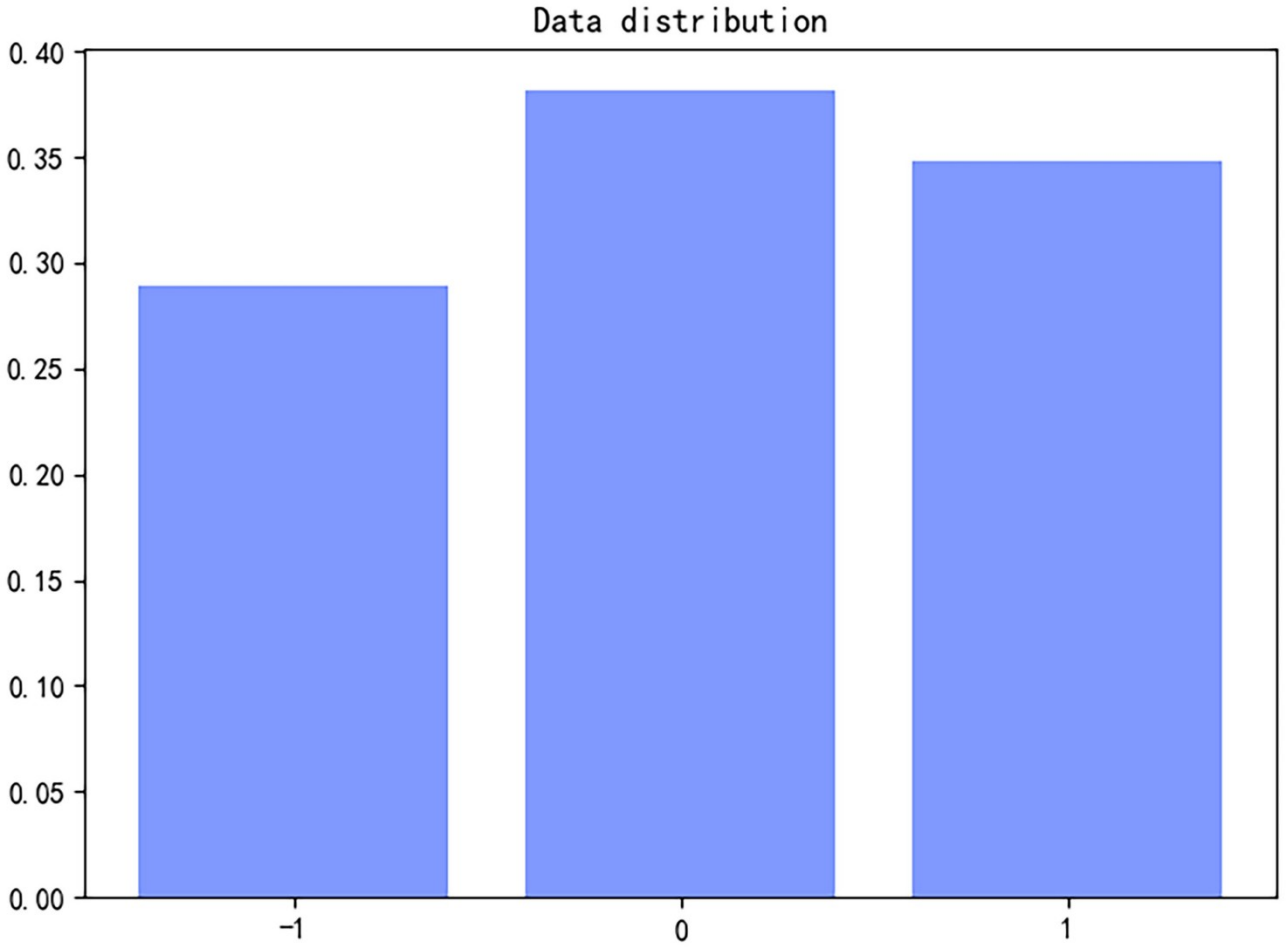
Data distribution. This picture shows the distribution of data in the dataset used in this article.

In this paper, the Dianping review dataset [8] from an assistant professor at Rutgers University is used to crawl and construct comments from a popular review website as well as a famous online review website in China, and then, users’ business scores are divided. Dianping.com divides the scores into 1, 2, 3, 4 and 5 levels. Through analysis of emotional tendency, this paper divides the emotional tendency into positive emotion and negative emotion. The comprehensive scores of 1 and 2 are divided into negative emotion, which is recorded as—1; the scores of 4 and 5 are divided into positive emotion, which is recorded as 1; and the score of 3 is regarded as the medium evaluation, which is recorded as 0. Although neutral evaluation has little effect on affective analysis, it can be used as the corpus for the training corpus model. This paper uses 250000 unlabeled comments and 300000 tagged comments, and the data distribution is shown in Fig 7.

In the experiment, the data used jieba word segmentation for standardized word segmentation, and the Baidu stop word list was used for preprocessing to remove stop words. However, this article screens the stop word list according to the actual situation and keeps some emotional words, such as good, very good, bad, dissatisfied and other words with a certain emotional tendency, which is helpful for text training.

**Fig 8:**
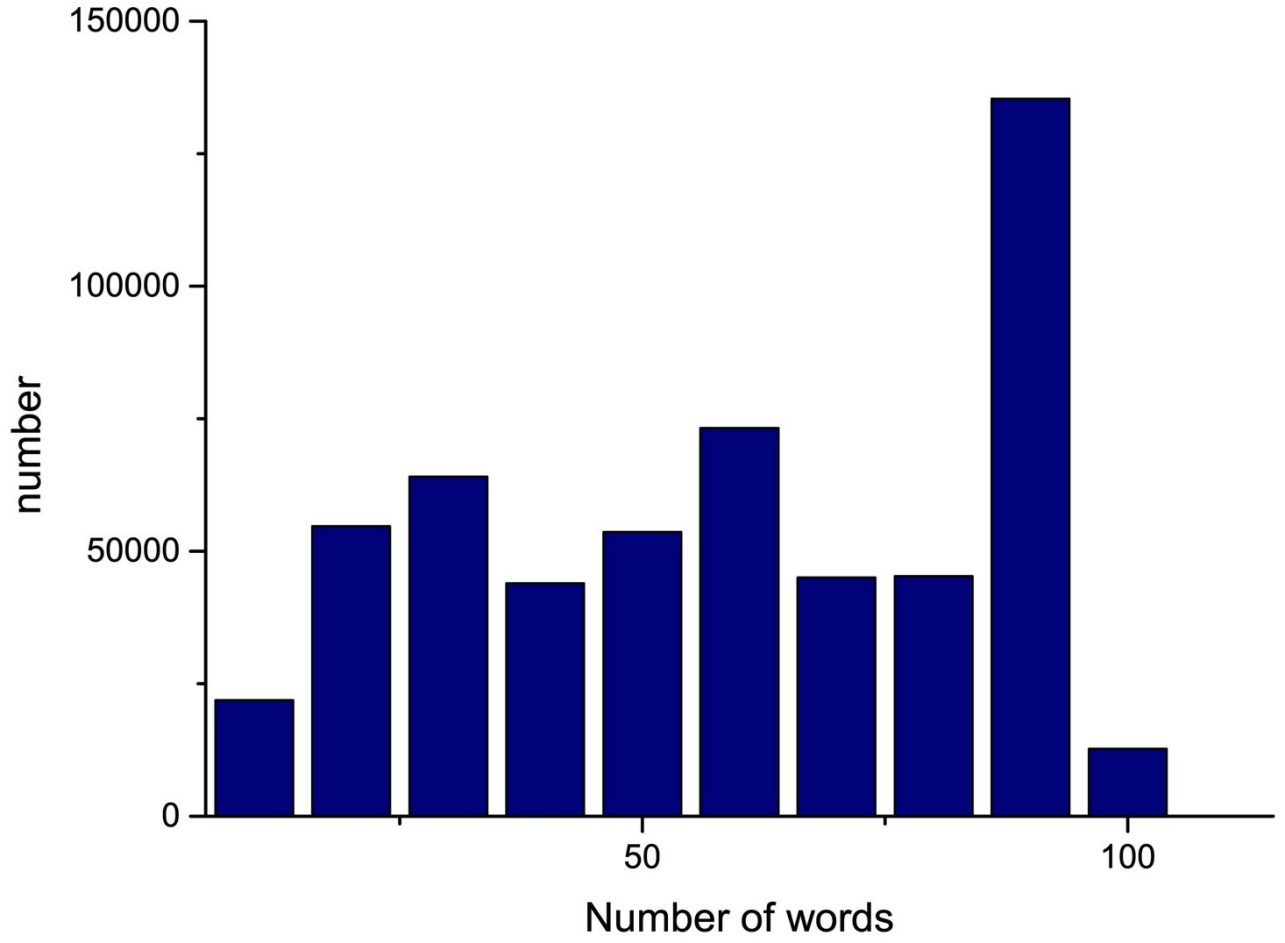
Quantitative distribution of words. This picture presents the number of words contained in each text in the dataset used in this paper.

Through the analysis of the data, as shown in Fig 8, it is found that the sentence length of 100 words can cover most of the data, so the sentence length input by the model is vectorized into 100 words, and the data set is divided into a training set and a test set according to the ratio of 80% and 20%, respectively. Examples of the data are shown in Table 1.

**Table 1:** Data sample.

Dataset	Models
1	Nanxin can be regarded as the famous dessert shop in Guangzhou. There are many people passing by for several time periods. Looking at the dense and delicious menu, it is easy to choose difficult diseases. Quickly withdrew after ordering. I went to the checkout counter and ordered. The waiter just served the food. The food tasted good.
2	Ordered red bean taro and coconut juice papaya red bean soup, sweet and greasy, canned coconut juice with coconut juice, a thick flavor of flavor, this shop can only be said to be overdone, and there are desserts in front Women are disgusting with a stinking face.

Part of the data in the data set.

### Experiment result and analysis

In the experiment, the number of layers of transformer is set to 1, the number of multihead attention for each encoder is set to 8, and other parameters are set according to Table 2 as follows. Three different evaluation indicators, including precision, recall and the F1 value, are applied to measure the quality of the model. Precision refers to the proportion of TP (True Positives) in all the elements labeled as belonging to the positive class (including TP and FP), recall is the percentage of TP in all the elements that actually belong to the positive class (i.e., the sum of TP and FN), and the F1 value is the harmonic average of precision and recall, and their formulas are as follows.

$$\begin{array}{c} \text{Precision}=\frac{\text{TP}}{\text{TP}+\text{FP}} \end{array}$$ (14)

$$\begin{array}{c} \text{Recall}=\frac{\text{TP}}{\text{TP}+\text{FN}} \end{array}$$ (15)

$$\begin{array}{c} F_{1}=\frac{2*\text{Precision}*\text{Recall}}{\text{Precision}+\text{Recall}} \end{array}$$ (16)

**Table 2:** Parameter setting.

Valuel	Value
Embedding dimension	100.000
Learning Rate	0.001
Dropout in Multi-Head Attention	0.9
SentenceLength	100.000
BatchSize	128.000

Model parameter settings.

**Fig 9:**
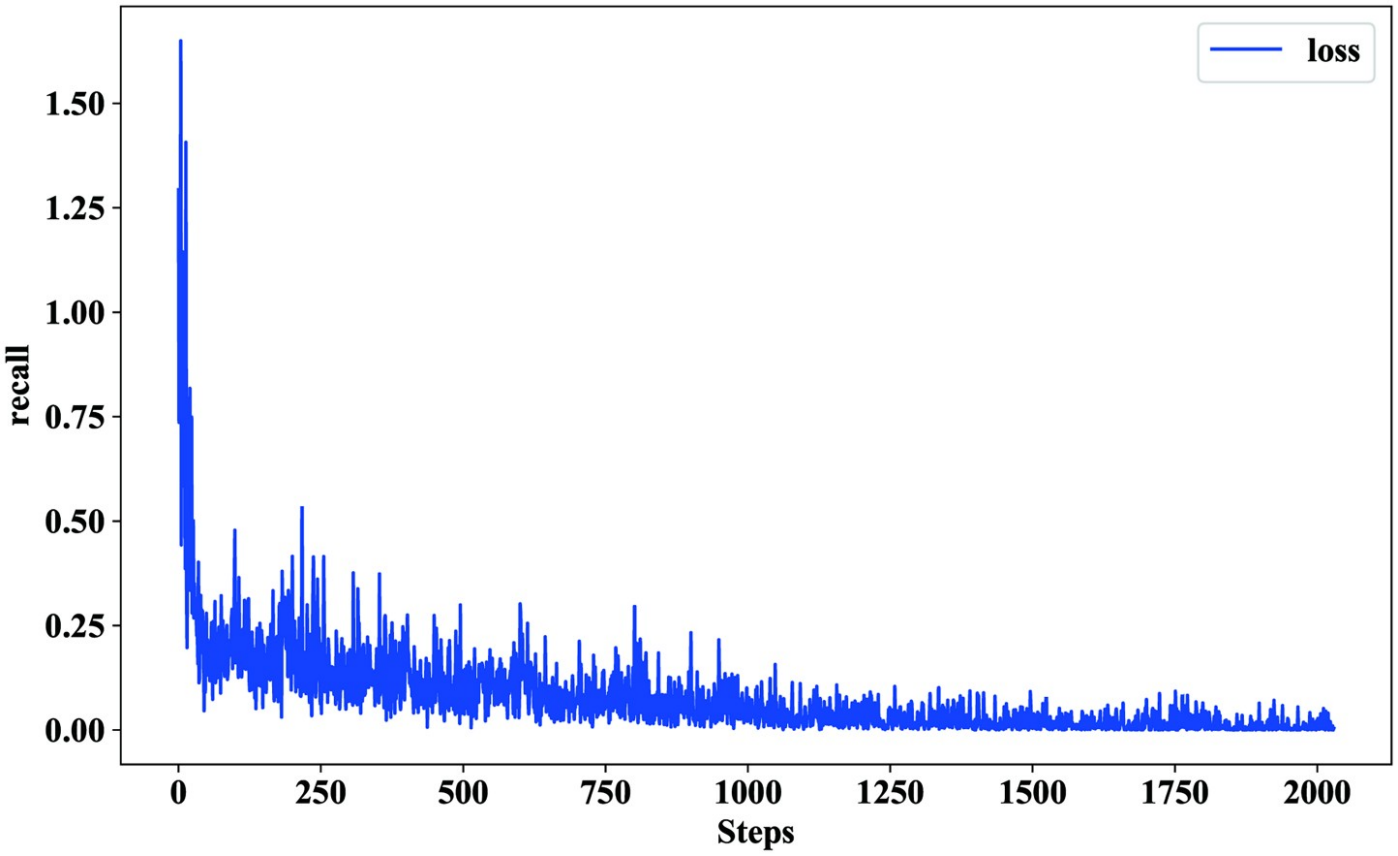
Changing curve of loss. This picture shows the change curve of the loss value of the model during training.

This paper calculates the binary cross entropy loss to evaluate the value of model loss. In the training process, it is found that the loss value decreases as the number of iterations increases. As shown in Fig 9, the convergence speed is very fast in the first 250 steps, as the loss value changes significantly. However, in 250-1500 steps, the convergence slows gradually, and the changing rate of the loss value declines. After 1500 steps, the loss value stabilizes.

**Fig 10:**
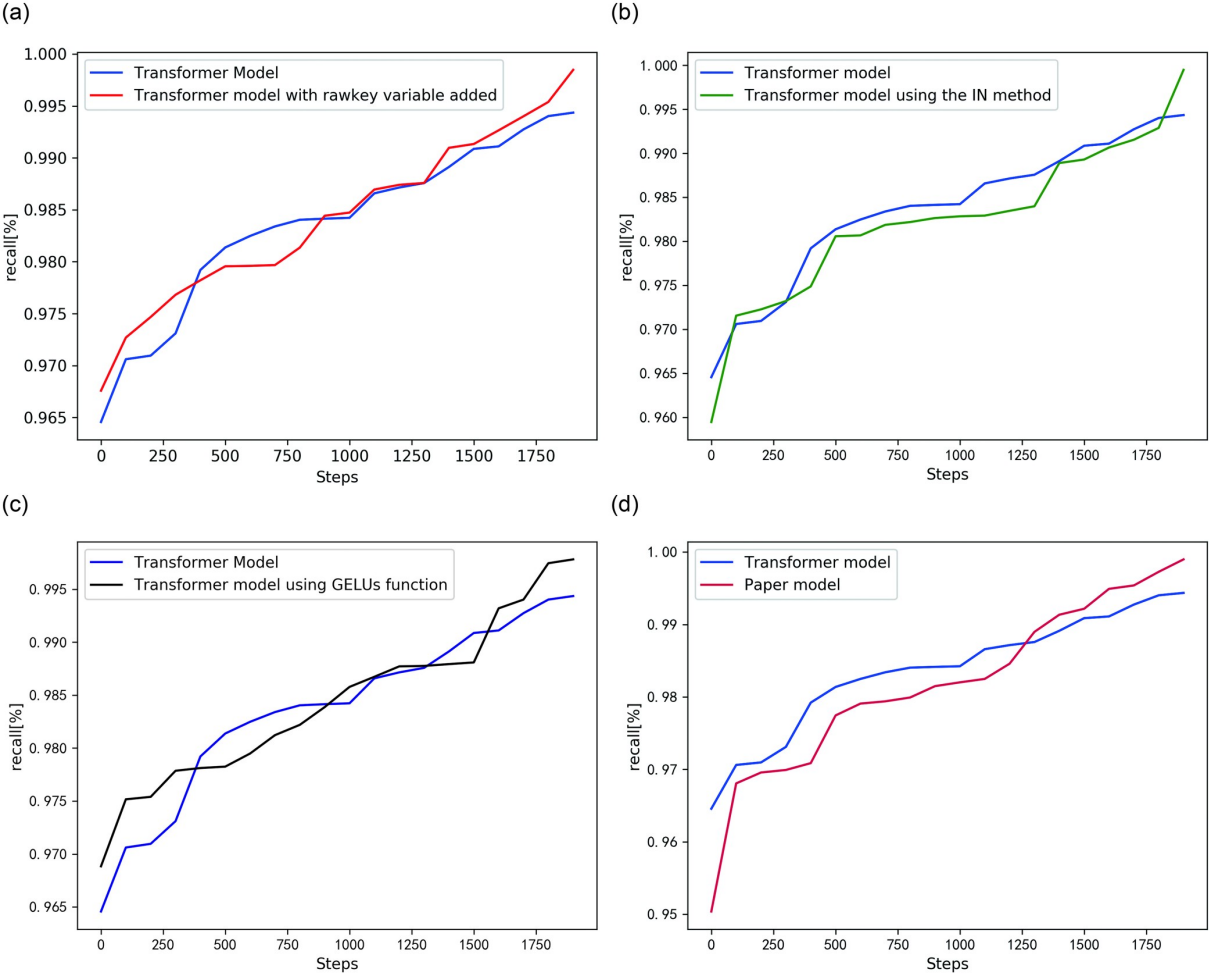
Comparative experiment of transformer model and improved model. (a) Increase rawkey variable; (b) Use the IN method; (c) Using the GELUS function; (d) Comparison of improved models.

As seen from Fig 10, this paper improves the original transformer model. When only adding a rawkey variable, the recall rate is improved by 0.4% compared with the original model. When only changing the IN method, the recall rate is increased by 0.51%. When only using the GELUs activation function, the recall increases by 0.38%. In general, this paper improves the original model, and the recall increased by 0.56% compared with original model.

**Fig 11:**
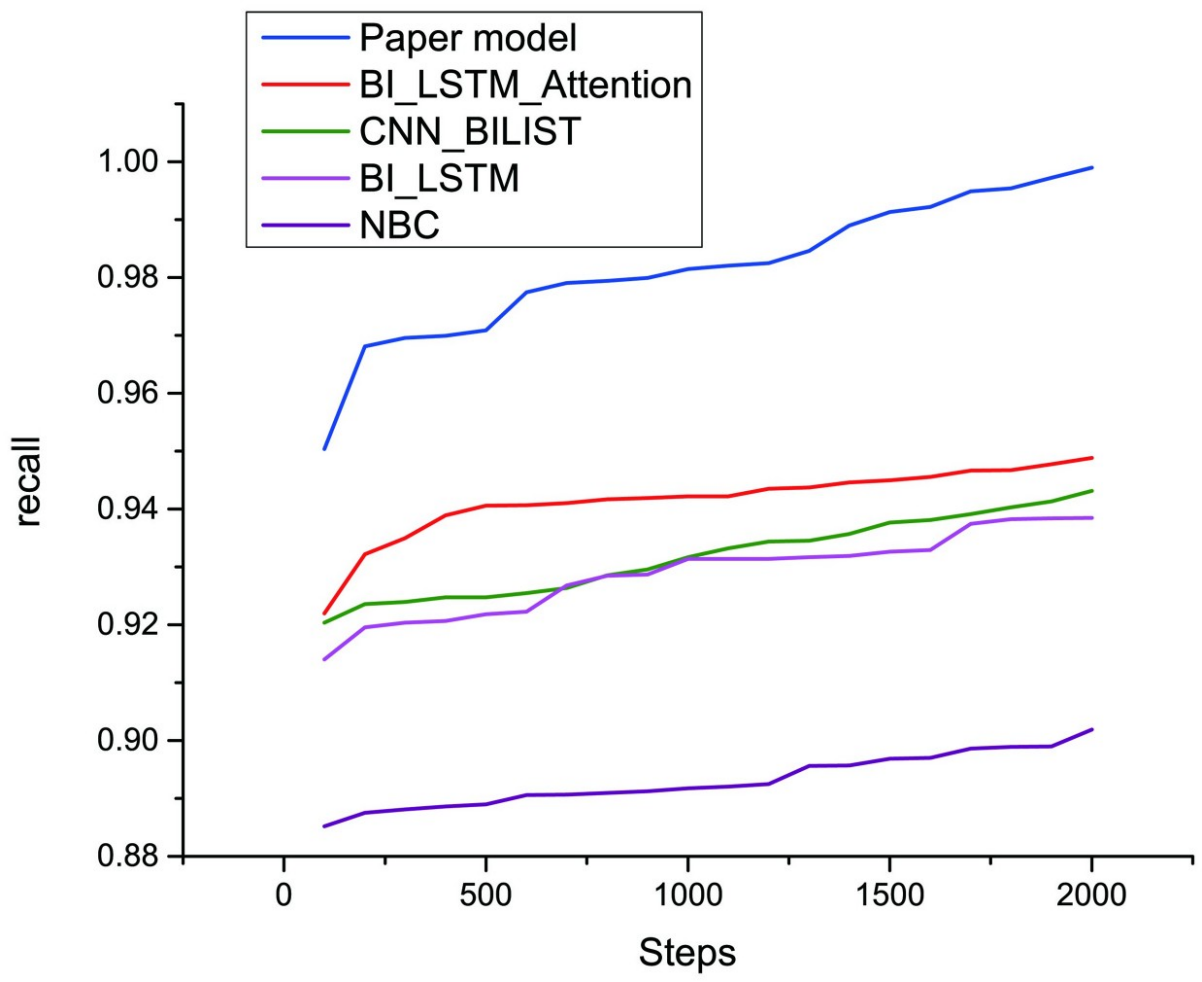
Model recall ratio comparison. This picture shows the change curve of the recall value compared with the two comparison models.

To verify the feasibility of this paper, this paper selects several relatively new text sentiment classification models to conduct a comparative test on the same dataset. The experimental results are shown in the following Fig 11 and Table 3.

**Table 3:** Experiment results.

Modell	Precisionl	recalll	*F*_1_value
NBC	0.8924	0.9019	0.9136
BILSTM	0.9186	0.9189	0.9574
Serial BiLSTM_CNN	0.9195	0.9224	0.9578
Bi_LSTM+Attention	0.9709	0.9478	0.9589
Our Model	0.9605	0.9989	0.9671

Comparison of experimental results.

Naive Bayesian classifier(NBC) [9]. The training samples are obtained by pre-processing the data, then estimating the probability of occurrence of different categories, and then the probability of occurrence of each attribute value under the condition of each category without sharply, so as to calculate the probability of belonging to each category for each combination of attributes, and finally selecting the maximum probability value as the output of the inferred result for that piece of data.

BiLSTM [10] is an improved version of LSTM by combining the forward LSTM with the backward LSTM to form the BiLSTM model. BiLSTM can better capture the semantic dependencies in both directions. For the sentiment classification task, the information obtained is more comprehensive, including all the information in the forward and backward directions.

Serial BiLSTM_CNN model [11]. BiLSTM is applied to extract context from text to resolve the problems of gradient disappearance and long-term dependence. Additionally, a CNN (convolutional neural network) is added to extract local semantic features from text, and softmax is used for processing at the last stage.

Bidirectional long short-term memory network with attention mechanism (BiLSTM+Attention) [12]. An attention mechanism based on the BiLSTM model is added; thus, self-attention weights are added to the text features of the current word. Then, the results are normalized through the softmax layer. The fully connected layer is utilized to output the processed matrix with attention weights.

By comparing the above 3 indicators, the modified transformer model proposed in this paper performs the best in text sentiment classification. A significant improvement is seen in both recall and the F1 value in comparison with BiLSTM, Naive Bayes Classifier, the serial BiLSTM_CNN model and BiLSTM with an attention. Based on the formulas for calculating precision and recall, only when these two values are both relatively large is the performance at this time optimal. Moreover, the F1 value can be regarded as a harmonic mean of these two values, allowing a comprehensive assessment of the quality of the model. It is found that when comparing the values of the 3 indicators, the precision and recall of the NBC model are both the lowest. BiLSTM+Attention enhances the acquisition of text features in current words by introducing the attention mechanism, so its precision is the highest among the five models. And the model used in this paper improves on the transformer model, which has the highest recall rate and the best F1 value for its overall evaluation.

**Fig 12:**
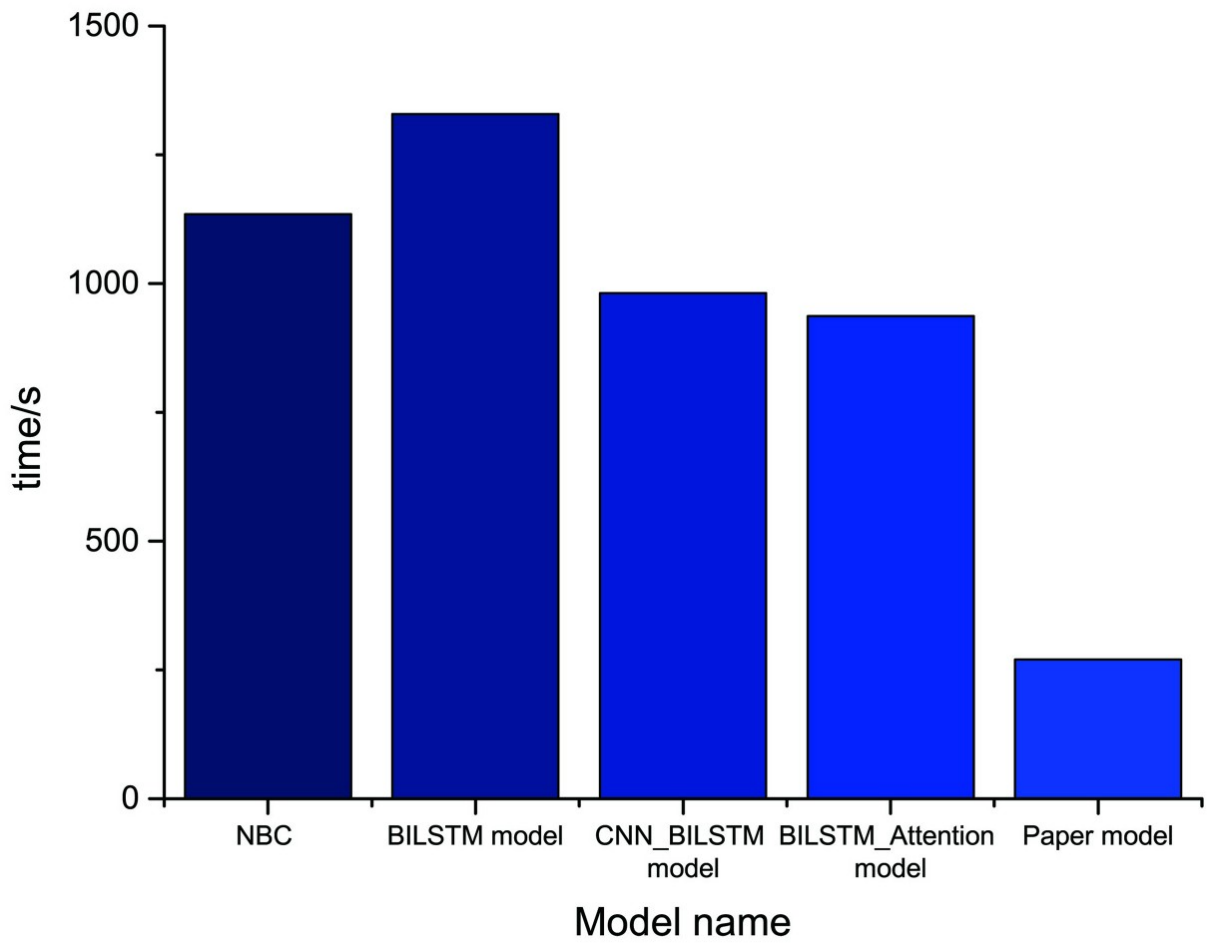
Comparison of model digestion time. This picture shows the time spent training the model compared to the two comparison models.

In terms of the time spent in model training, as shown Fig 12, the training time of the modified model in this paper is far less than that consumed by NBC, BiLSTM, serial BiLSTM_CNN and BiLSTM+Attention, decreasing the results by4.19 times, 4.91 times, 3.55 times and 3.38 times, respectively.

The text sentiment classification model based on the modified transformer proposed by this paper performs the best in terms of recall rate. In comparison with the original BiLSTM+Attention, its recall increases by 5.12%. Taken together, the F1 value of our model is the highest among the 5 models due to the following reasons. 1. IN is applied as the normalization method in the model. The advantages of IN are used to reduce the impact of channels and batchsize on the model. 2. A new activation function, GELUs, is used, and the random regularization idea is utilized in the process of activation, which is intuitively more in line with natural cognition. 3. The variable rawkey is added in multihead attention, and the data are optimized and completed before the softmax application, which is beneficial to the optimization of the model. 4. As for the dataset, e-commerce reviews have a tight context, which highlights the advantages of the transformer model. Through the above analysis of the experimental results, it is demonstrated that the modified text sentiment classification model based on transformer proposed in this paper performs better than the serial BiLSTM_CNN and BiLSTM+Attention models, and the experimental results verify the effectiveness and feasibility of our method.

## Conclusion

This paper proposes a text sentiment classification model using e-commerce reviews as the basis of an advanced transformer. A new variable, rawkeys, is added to multihead attention to make the model more accurate when processing data. In addition, a new normalization method, IN, is utilized to process the data results. A new activation function, GELUs, is applied at the end, which enhances the generalization ability of the whole model, makes the process more in line with the natural cognition process, and further shortens the time required for model training. The weaknesses of our model are that it takes too long to train data of a large volume, it is not optimized well for preprocessing data, and it does not perform as well as the BiLSTM + Attention model in terms of precision. In future work, the model will be improved, the data at the preprocessing phase will be optimized, and the advantages of other models will be integrated to improve the classification performance of this model, guaranteeing stable recall.
